# Judicious Transfusion of Platelets Among Neonates: A Systematic Review

**DOI:** 10.7759/cureus.65818

**Published:** 2024-07-31

**Authors:** Saritha Kamsetti, Saritha Tammali, Ashok Kumar Urakurva, Rakesh Kotha

**Affiliations:** 1 Pediatrics, Government Medical College, Vikarabad, Vikarabad, IND; 2 Pediatrics, Niloufer Hospital, Hyderabad, IND; 3 Neonatology, Osmania Medical College, Hyderabad, IND

**Keywords:** neonatal hemorrhage, transfusion-related reactions, thrombocyte, platelet-rich plasma/prp, "neonate"

## Abstract

In newborns, especially premature babies, there is a high association between thrombocytopenia and bleeding, particularly intraventricular hemorrhage, which may be due to immaturity. It was usual clinical practice that neonates should be transfused with higher platelet counts than older children or adults to reduce their risk of bleeding. However, after keen observations, we noticed that bleeding and mortality were more common in newborns who received more platelet transfusions. The mechanisms underlying the adverse effects of platelet transfusions in neonates may be due to higher antigenicity and immunological factors. We know that neonatal platelets are hyporeactive; this hyporeactivity is balanced by factors in the neonatal blood that promote coagulation, such as increased hematocrit, von Willebrand factor, and fibrinogen, which, on balance, leads to normal primary neonatal hemostasis. Platelets are very similar to adults in number, but functional capabilities were less, and for the reasons mentioned above, particularly bleeding time was short. Theologically, neonatal platelet lifespan was high to compensate for less production. We started this review because we observed that many babies were not having bleeding symptoms in some instances of severe thrombocytopenia. Many well-active babies are receiving unnecessary transfusions, as human blood is precious, and many young neonatologists are going on protocol-based excessive transfusions. This stimulated us to write a review.

## Introduction and background

Platelet transfusions are a crucial part of care in neonatal intensive care units (NICUs). They are often given to stop bleeding complications in preterm babies and neonates with thrombocytopenia [1]. However, there is a growing concern today about the overuse of platelet transfusions and the possible adverse effects related to them. Such impacts include alloimmunization, transfusion reactions, and increased morbidity. Recent studies have suggested that many neonates requiring platelet transfusions improve spontaneously without intervention [2]. It raises some fundamental questions regarding whether the procedures are necessary or needed as frequently. Good judgment in platelet transfusions involves balancing the benefit of preventing severe bleeding with the risk and expense associated with unnecessary transfusions. The systematic review critically evaluates the evidence about the need for judicious platelet transfusions in a newborn ICU. The review will outline current practices and recommendations for using the synthesized data from several studies and the optimal use of platelet transfusion protocols in neonates. It would also help lay the foundation for informing clinical guidelines and improving patient outcomes through the appropriate and effective use of platelet transfusions in neonatal populations.

## Review

Search strategy

Multiple databases have been searched for relevant studies regarding platelet transfusions in neonatal ICUs. Such databases included PubMed, the Cochrane Library, and Scopus. The combination of Boolean operators with terms such as "platelet transfusions," "NICU," "neonatal intensive care unit," "unnecessary transfusions," and "judicious use" was put into practice while searching (Table 1). Such a strategy should retrieve studies that are either randomized controlled trials (RCTs), systematic reviews, or cohort studies. References to relevant articles were hand-searched to identify any missing researchers during the process. We registered under the International Prospective Register of Systematic Reviews (PROSPERO) with ID 568854.

**Table 1:** Search strategy used for systematic review NICU: neonatal intensive care unit

Serial number	Search terms
#1 Search	Platelet transfusion [Mesh], NICUs [Mesh], thrombocytopenia [Mesh]
#2 Keywords search: title/abstract	Platelet transfusion in NICUs, neonatal platelet transfusion practices
#3 Keyword search: truncation	Platelet transfusion AND NICU, transfusion complicated" AND neonate
#4 Keyword search: alternative combinations and terms	Neonatal care AND platelet transfusion guideline NICU practices AND platelet transfusion, premature baby AND platelet transfusion risks
#5 Keyword search: broader terms	Infant blood transfusion, neonate transfusion safety, newborn intensive care transfusion

Inclusion and exclusion measures

**Figure 1:**
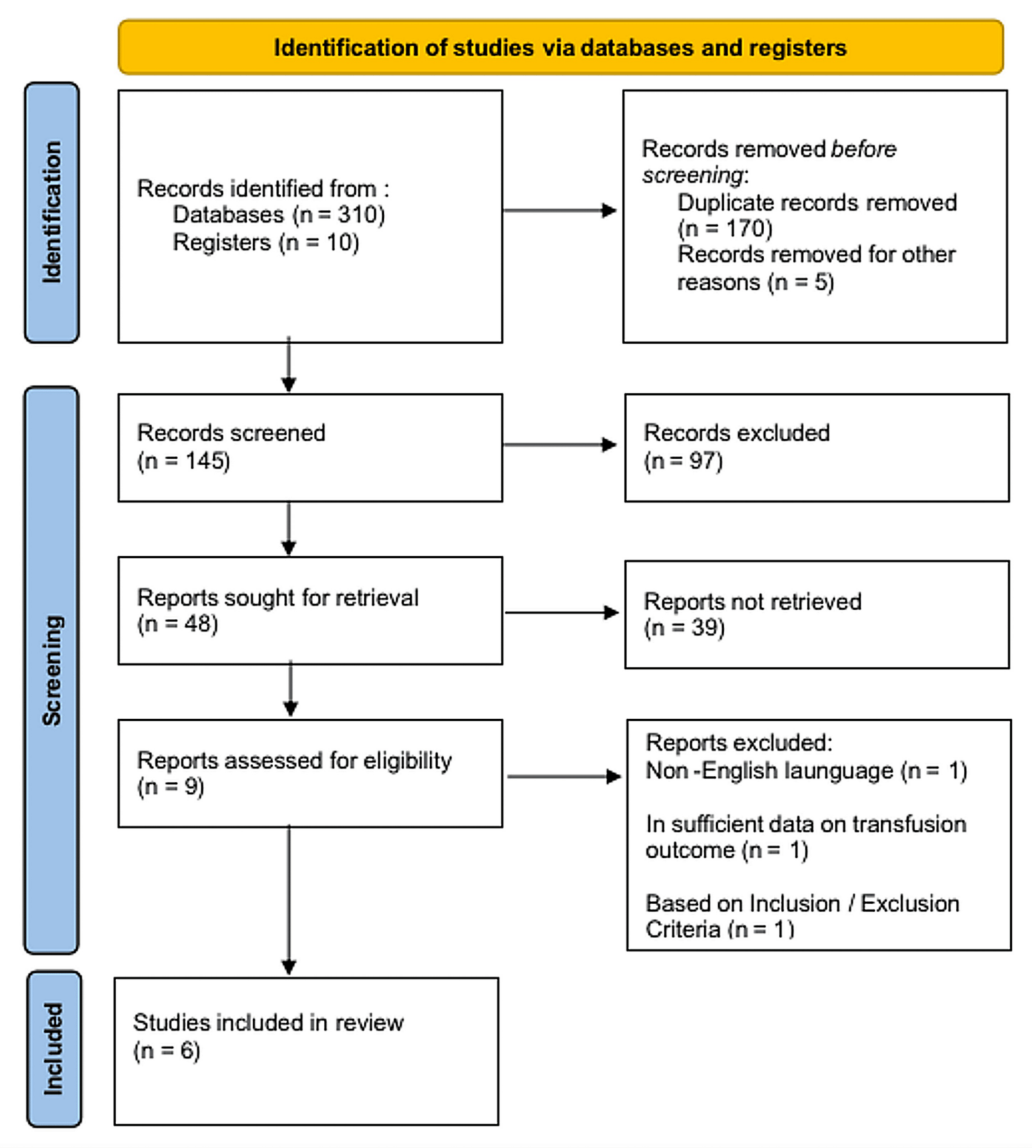
PRISMA flow diagram showing the study identification and selection process PRISMA: Preferred Reporting Items for Systematic Reviews and Meta-Analyses

The different studies had to meet the inclusion criteria to be enlisted for the review. Studies were eligible if they involved platelet transfusions in preterm infants and term neonates, more so on the necessity and outcomes of such transfusions. The excluded studies did not share the necessity of transfusions or their significance. Studies published in English were considered for easy accessibility and understanding of information. Researchers excluded articles written in other languages other than English. RCTs and meta-analyses are sought after more because of the high level of evidence associated with them. Studies that were based on subjective research approaches were then not considered. Exclusion criteria were also set to ensure there was focus in the review and that the included studies were well designed. Such a rigorous selection process was intended to reduce bias and allow for the inclusion of high-quality evidence relevant to the research question. The systematic review was done in compliance with the Preferred Reporting Items for Systematic Reviews and Meta-Analyses (PRISMA) guidelines (Figure 1).

Data extraction and quality assessment

Two reviewers did data extraction independently to minimize errors and reduce bias. Extracted key data elements include study design, population characteristics, interventions, and critical findings. Discrepancies between reviewers were leveled out through conversation and agreement. The evaluation relied on the Newcastle-Ottawa Scale (NOS), effectively determining the quality of observational studies. The evaluated studies were marked as high quality based on the NOS scale. Studies that were completed using RCTs were assessed using the risk of bias 2 (ROB2; The Cochrane Collaboration, London, England, UK) approach. Researchers had to answer different questions when using ROB2 to ensure they assessed every resource effectively. The questions were based on the study’s interventions, the randomization process, and data regarding the results. Researchers marked the articles as low-bias based on the ROB2 scale. Researchers also used AMSTAR, which is an effective tool for evaluating studies completed using systematic reviews. The major aspects that were considered during the evaluation included the type of resources utilized and how authors synthesized results. The assessed studies attained a high score, as they all gained good grades. Researchers had eliminated the chances of bias in their outcomes and cohort groups as evaluated using NOS. The score indicated the need to progress with the chosen articles to research the main topic. Table 2 contains summaries of selected studies.

**Table 2:** Summary of the studies included in the systematic review

Study	Study type	Year	Country	Objective	Comparative group	Conclusion/outcome
Curley et al. [3]	Randomized controlled trial	2019	England	To provide evidence concerning the thresholds associated with conducting platelet transfusions	Preterm infants	Defined transfusion thresholds that reduce mortality without increasing risk
Davenport et al. [4]	Cohort study	2024	The United States	Examine platelet transfusion mechanisms and approaches	Standard treatment	Association of transfusions with mortality and neurodevelopmental impairment
Liu et al. [2]	Systematic review protocol	2020	China	To assess issues associated with blood transfusion among neonates	Platelet transfusion among patients with thrombocytopenia	Protocol development for systematic review on neonatal thrombocytopenia
Moore et al. [5]	Randomized controlled trial	2023	Ireland, the UK, and the Netherlands	Examine mortality and neurodevelopmental issues among neonates who underwent transfusion	Standard treatment	Improved neurodevelopmental outcomes at two years post-transfusion
Ribeiro et al. [1]	Systematic review and meta-analysis	2023	Europe	To explore facts concerning platelet transfusion outcomes	Non-transfused infants	Identified controversies in current transfusion practices
Sola‐Visner et al. [6]	Randomized controlled trials	2022	The United States	The study assesses the effectiveness of conducting platelet transfusions among neonates	Adult platelets	Challenges in translating evidence-based recommendations into practice

Description of studies

Moore et al. assessed the platelet transfusion thresholds in preterm infants. The completed PlaNeT-2/MATISSE trial has reported several outcomes over the past two years. About 48% of infants in the higher platelet transfusion threshold of 50×10^9 /L had death or significant neurodevelopmental impairment, compared to 38% in the lower threshold of 25×10^9 /L [5]. Such a critical finding raises concerns about the possible dangers of higher levels of blood product transfusion in the population. The adverse outcomes affected 50% of infants in the higher threshold group compared to 39% of infants in the lower threshold group, which translates into an odds ratio of 1:54 in favor of the lower threshold group [5]. Such long-term, multi-site research in the UK, Netherlands, and Ireland NICUs from 2011 to 2017 strengthens clinical practice to adopt safe transfusion practices.

The systematic review protocol from Liu et al. focuses on finding platelet transfusion thresholds for neonates with thrombocytopenia encountered in the NICU [2]. It increases the bleeding and mortality rates. Some coherent procedures they suggested were the Cochrane Handbook and guidelines, the PRISMA statement, and the GRADE approach. The review will include only RCTs and comparable studies, such as cohort and case-control, depending on factors like in-hospital mortality and bleeding episodes. Data from the first cross-sectional survey indicates a divergence in transfusion practices among NICUs, and the current guidelines are not supported strongly by evidence. The review results will help clarify clinically relevant platelet transfusion triggers and safety concerns that might help avoid unnecessary platelet transfusions in neonates with thrombocytopenia. Besides, it will also ensure early treatment and minimize the risks of adverse effects.

Ribeiro et al. mentioned that platelet transfusions in premature infants with thrombocytopenia have associations with adverse effects. The present research identified that platelet transfusions boosted mortality and morbidity, including sepsis and necrotizing enterocolitis (NEC), in preterm neonates. According to the meta-analysis, platelet transfusion was associated with an increased mortality rate (RR 2. 4), sepsis (RR 4. 5), and NEC (RR 5. 2). However, it was moderately associated with intraventricular hemorrhage (IVH). The relationship was not statistically significant because the trials were highly heterogeneous. Such findings support the notion that platelet transfusion is a risky procedure for preterm infants and should be used sparingly. Therefore, research needs to be done on safe transfusion thresholds to avoid the adverse effects of the interventions [1].

In a cohort study of extremely preterm infants by Davenport et al., platelet transfusion was disproportionately linked to higher rates of mortality. It also entailed severe neurodevelopmental issues (NDI) at two years of corrected age. The proportion of infants experiencing the primary outcome was 46.5% among the neonates who underwent platelet transfusions during NICU hospitalization. Even after controlling for confounding factors like gestational age and trial treatment group, an adjusted odds ratio of 2.43 demonstrated the evidence of the association. Additional analysis showed that with each additional platelet transfusion, there was a significantly 25% increased likelihood of death or severe NDI. The second outcome also suggested negative impacts in the process. For instance, post-transfusion infants had lower motor scores than the no-transfusion group. Such observations raise the possibility that platelet transfusion may be associated with harm to the population. It raises the question of when and whether platelet transfusions should be initiated and maintained with an eye on developmental outcomes [4].

The study by Curley et al. on the effects of platelet transfusion did not observe differences in the primary endpoint of death or significant bleeding. It was observed on the 28th day between the low-platelet-transfusion threshold group of fewer than 25,000 platelets per cubic millimeter and the high threshold group of below 50,000 per cubic millimeter. However, additional babies in the high-threshold collection received platelet transfusions unrelated to decreased mortality. The other secondary findings of mortality rates with bronchopulmonary dysplasia and significant bleeding episodes were also observed to be insignificant within the two groups. Such observations imply that a lower platelet transfusion threshold offers no additional clinical advantage over the RCT of mortality in preterm infants with severe thrombocytopenia [3].

Current research shows that although platelet transfusions are commonly used in NICUs, their necessity and effectiveness are controversial. Prophylactic transfusion does not reduce the risk of bleeding in infants without bleeding. The study highlights the threshold for transfusion and the need for greater selection. We are not done with meta-analysis due to the huge heterogeneity.

Discussion

Balancing the appropriate prescription of platelet transfusions in NICUs is essential to prevent adverse effects from transfusion while achieving a good outcome for the patient [7]. The discussion brings out the systematic review's findings on the original question of unnecessary transfusions in NICUs. The systematic review outlined the dangers and opportunities of platelet transfusions in NICUs. It established several issues concerning platelet transfusions in NICUs. For instance, if physicians infuse excessive platelets, the child will likely experience a sharp rise in blood volume, which can lead to high pressure [6]. Additionally, neonates are likely to experience inflammation during transfusion, indicating the need for intense care to prevent the development of other conditions [8]. Moore et al. and Curley et al. wrote about different transfusion thresholds and their outcomes [5,3]. The systematic review also involved meta-analyses that analyzed pooled data to identify tendencies for transfusion in variable NICU facilities [1].

An essential strategy for optimizing platelet transfusion observed in neonatal ICUs is the practice of patient- or family-centered care, focusing on an individual's needs. Notably, NICU practitioners can achieve holistic care goals by centering their care practices on patients [9]. Premature infants show a great deal of variability in clinical conditions and responses to treatments. An action may be taken by connecting primary genetic and clinical data on personalized medicine. Notably, personalized medicine within neonatal units and facilities would require caregivers to rely on genetic data to diagnose and treat patients [10]. For instance, perceiving the risk profile for thrombotic complications at the individual infant level will aid in making more rational decisions. Data from neonates is also crucial, considering that they are more susceptible to bleeding [11]. Therefore, engaging parents and other caregivers in the decision-making process at different levels assures the respect of values and preferences through partnership in care [12].

The findings have important implications and present a complex picture of transfusion decisions. The key factors that must be considered when determining the possible complexities include postnatal age and other physiologic differences [13]. Early platelet transfusions may be effective in preventing IVH in infants weighing under 1500 g. Blood transfusion specialists should be concerned about neonates with a platelet volume that does not surpass the < 100 × 109/L limit, as it is a potential range for IVH [14]. Elgendy et al. support that determining the required volume is crucial because administering more platelets is associated with excessive bleeding [15]. Therefore, delays and inaccuracies in performing transfusions pose a significant threat to neonates as they expose them to critical conditions. Neonates are also exposed to fungal infections, including Candida, during transfusion [16]. Neonates may also be diagnosed with cytomegalovirus, a critical infection contributing to worse states of thrombocytopenia [17]. Further research indicates that newborns are subject to transfusion reactions associated with allergic conditions, which can lead to distress during transfusion [18]. Unfortunately, the criteria for starting platelet transfusions are still contentious [4]. It calls for formulating protocols that incorporate clinical markers and patient-specific attributes. Specialists must also develop effective approaches to challenging situations, such as refractoriness, and address concerns about blood type compatibility [19]. Refractoriness can be a critical concern among specialists as the issue is induced by nonimmune conditions, including medications and bleeding [20].

The systematic review approach posed two limitations to the current research. The first limitation encompassed the credibility of the utilized articles despite conducting an assessment. The current study was of low quality as it relied on previously completed studies, which might not have been authentic or grounded in high-quality approaches. Much time was also spent acquiring high-quality resources for the study. The second limitation was that the review did not allow researchers to expand to other correlated topics that would help understand the main issue. Researchers had to focus on the research question that guided the review. Lastly, researchers were challenged by the different research methods each article applied to develop a specific conclusion. The utilized resources did not all have a similar methodology, sometimes making the analysis complex.

Recommendations

Healthcare professionals should establish guidelines regarding platelet transfusions in NICUs across different centers. Such protocols should be derived from research and depend on gestational age and birth weight. It would also entail other clinical indications for initiating transfusions. Potential variations in the practice can be minimized for maximum effectiveness, and blood transfusions can be delivered only when required. However, more research should be undertaken to evaluate the risk factors before administering platelet transfusions to premature infants. Such assessment should involve weighing the potential benefits of preventing conditions like IVH against complications due to transfusion-borne infection risks. The approach ensures that the process of consideration for transfusion is done based on the patient’s needs.

Adequate monitoring and surveillance activities need to be implemented to monitor the results related to PT in NICU environments. Children’s neurodevelopment up to school age, as well as transfusion-related complications, should be documented and evaluated. Ongoing data collection and analysis support the refinement of quality improvement initiatives and allow healthcare providers to modify transfusion practices based on current results.

Various stakeholders should develop close cooperation between neonatologists and transfusion specialists. Through teamwork and an interdisciplinary approach, the treatment teams can achieve the best results in transfusing the patient and enhancing the NICU settings. Neglecting the need for teamwork within neonatal care units is a primary cause of deaths and unsatisfactory outcomes [21].

Facilities can be funded to acquire blood system technologies that would assist in eliminating pathogens during transfusion [22]. Specialists also have the potential to innovate and apply strategies that integrate pathogen-reduction technology to promote the safety of neonates [23]. Further research should be done on other therapies and technologies that minimize exposure to platelet transfusion, such as recombinant thrombopoietin and platelet substitutes. Identifying other approaches to thrombocytopenia in prematurely born infants can help increase the range of available treatments and improve the quality of care in NICUs. For example, holistic care would have an impact on eliminating the distress associated with the condition. The kangaroo mother care approach can be examined, considering that the intervention has assisted in promoting neonatal health benefits such as reducing sepsis [24]. The current study also recommends deeper research into the most contradictory practices concerning platelet transfusion. Issues such as storage duration, inactivation procedures, and appropriate additives are prevailing concerns about platelet transfusion that should be addressed [25].

Healthcare institutions must incorporate standard operating procedures and research to support optimized transfusion practices. They should aim to improve individual medicine, as every baby is unique in its genetic and maternal environment. Kangaroo mother care and proper nutrition, particularly breastfeeding, will help in decreasing platelet transfusions.

## Conclusions

This systematic review fosters the complex landscape of platelet transfusion in neonatal ICUs. A synthesis of available literature underlines the variability in transfusion thresholds and practices across different settings within NICUs. It reflects the ongoing debate about using optimal transfusion strategies for premature infants. Therefore, the project incorporates standardized protocols steered by evidence-based guidelines and continued research, which are significant keys to improved decision-making in transfusion and subsequent outcomes for NICU populations. Healthcare providers should be set up to reduce avoidable transfusions. It requires advancing the profession toward safer, more effective transfusion practices within NICU settings. Collaboration among team members is required to ensure that pressing issues have been addressed. Thus, policymakers should ensure better protocols are followed in every healthcare facility for better patient outcomes while at the same time considering individual baby genetics and their mother environments.
